# Artificial Intelligence Technologies for Coping with Alarm Fatigue in Hospital Environments Because of Sensory Overload: Algorithm Development and Validation

**DOI:** 10.2196/15406

**Published:** 2019-11-26

**Authors:** Chrystinne Oliveira Fernandes, Simon Miles, Carlos José Pereira De Lucena, Donald Cowan

**Affiliations:** 1 Department of Informatics Pontifical Catholic University of Rio de Janeiro Rio de Janeiro Brazil; 2 Kings College London London United Kingdom; 3 University of Waterloo Waterloo, ON Canada

**Keywords:** alert fatigue health personnel, health information systems, patient monitoring, alert systems, artificial intelligence

## Abstract

**Background:**

Informed estimates claim that 80% to 99% of alarms set off in hospital units are false or clinically insignificant, representing a cacophony of sounds that do not present a real danger to patients. These false alarms can lead to an alert overload that causes a health care provider to miss important events that could be harmful or even life-threatening. As health care units become more dependent on monitoring devices for patient care purposes, the alarm fatigue issue has to be addressed as a major concern for the health care team as well as to enhance patient safety.

**Objective:**

The main goal of this paper was to propose a feasible solution for the alarm fatigue problem by using an automatic reasoning mechanism to decide how to notify members of the health care team. The aim was to reduce the number of notifications sent by determining whether or not to group a set of alarms that occur over a short period of time to deliver them together, without compromising patient safety.

**Methods:**

This paper describes: (1) a model for supporting reasoning algorithms that decide how to notify caregivers to avoid alarm fatigue; (2) an architecture for health systems that support patient monitoring and notification capabilities; and (3) a reasoning algorithm that specifies how to notify caregivers by deciding whether to aggregate a group of alarms to avoid alarm fatigue.

**Results:**

Experiments were used to demonstrate that providing a reasoning system can reduce the notifications received by the caregivers by up to 99.3% (582/586) of the total alarms generated. Our experiments were evaluated through the use of a dataset comprising patient monitoring data and vital signs recorded during 32 surgical cases where patients underwent anesthesia at the Royal Adelaide Hospital. We present the results of our algorithm by using graphs we generated using the R language, where we show whether the algorithm decided to deliver an alarm immediately or after a delay.

**Conclusions:**

The experimental results strongly suggest that this reasoning algorithm is a useful strategy for avoiding alarm fatigue. Although we evaluated our algorithm in an experimental environment, we tried to reproduce the context of a clinical environment by using real-world patient data. Our future work is to reproduce the evaluation study based on more realistic clinical conditions by increasing the number of patients, monitoring parameters, and types of alarm.

## Introduction

### Alarm Fatigue

Information Technology (IT) has already provided significant benefits to the health care sector, but there are still many areas where the application of IT could offer further critical improvements. For example, alarm fatigue, which has recently been receiving attention from industry, the health care sector, and the academic community, is a worldwide hospital problem.

Alarm fatigue involves a lack of response because of an excessive number of noncritical alarms being received by health care personnel, resulting in sensory overload and desensitization [1-4]. To illustrate the severity of this problem that has been treated as a major patient safety concern, scientific studies have reported that there was an average of 700 physiologic monitor alarms per patient per day [1]. Such a number indicates a severe sensory overload for the health care staff, with serious consequences for the well-being of the patients when an alarm might be ignored.

In this paper, we present a new approach to coping with the alarm fatigue problem, its most common causes, adverse consequences, and strategies as compared with other solutions published in the literature [5-9]. Our proposed solution for addressing this issue uses an artificial intelligence (AI) approach based on an automatic reasoning system that decides how to notify caregivers about anomalies detected by a patient monitoring system where a large volume of alarms could lead to alarm fatigue. In other words, we are using IT to reduce the number of notifications received by health care staff, so they can be focused on the activities that truly need attention. Our experiments were configured to alert nurses and were evaluated through the use of a dataset comprising a wide range of patient monitoring data and vital signs that were recorded during 32 surgical cases where patients underwent anesthesia at the Royal Adelaide Hospital [10].

In this work, we aim at addressing 2 main research questions: (1) How can an automatic reasoning system determine how to notify caregivers about anomalies detected by a patient monitoring system where a large volume of alarm leads to alarm fatigue? (2) How to reason about avoiding alarm fatigue?

Our main goal with this case study is to find out whether to group a set of alarms that happens within a short period of time to deliver them together without compromising patient safety. Our specific goal is to avoid that alarms of the same type for the same patient can be alerted more than once within a short period by using a notification delay strategy.

### Theoretical Background

#### Related Work

A critical concern in hospitals that use monitoring devices to track patients’ health is alarm fatigue. Tens of thousands of alerts may go off throughout a hospital each day, and yet some 80% to 99% of these audible or visual alerts are false or nuisance alarms, indicating conditions that do not require clinical intervention [1-4]. Alarm fatigue represents a substantial issue that can bring undesired consequences to health care environments. For instance, the desensitization of a health care team to alerts can lead to longer response times for handling anomalies as well as possibly missing life-threatening events. These examples illustrate the fact that sensory overload is very likely to produce an unsafe environment for patients.

According to Sowan et al [6], the key issues causing alarm fatigue and reducing trust in alarm systems are as follows: the high incidence of nuisance alarms, the confusion in locating the device sending out the alarm, unit layouts that hinder alarm response, the inadequacy of alarm systems to alert nurses of changes in patients’ conditions, and the complexity of new monitoring systems, among others. The most important issues interfering with alarm recognition and alarm response ranked by the nurses in [6] were as follows: (1) frequent false alarms, (2) difficulty in understanding alarm priorities, and (3) noise competition from nonclinical devices.

Caring for patients and managing alarms simultaneously is a very complex and demanding task, especially when health providers are caring for multiple patients at the same time and have been exposed to a high number of alarms generated by physiological monitors. In addition to dealing with frequent alarms, health care providers also perform other activities, such as medication administration, patient assessments, and note updates. Over time, they become fatigued and errors may occur because of decreased attentiveness [5].

Considering the aforementioned scenario, a commonly recommended solution to mitigate alarm fatigue is to adjust alarm parameters on monitors to suit each patient’s conditions rather than using default settings [5]. The works of Shanmugham et al [5] and Sowan et al [6] are examples of studies that assess the effect of modifying the default alarm settings provided by the device manufacturers. According to their findings, the nurses’ perceived workload was lower when the clinical alarm threshold limits were modified according to patients’ clinical conditions. They also concluded that the modification of alarm settings affects the number of alarms accurately addressed, care providers’ experience, and overall satisfaction.

Another strategy suggested to reduce the number of false alarms and alarm fatigue is educating staff regarding alarm management [6]. Sowan et al showed that their changes in default alarm settings significantly reduced 24% of the total number of the target alarms after their interventions, which included the following: (1) re-education of intensive care unit (ICU) bedside nurses on the appropriate use of the monitors, and (2) changing default settings of some parameters on the cardiac monitors, including the addition of an alarm delay by increasing the period between the alarm detection and its triggering, among others.

However, despite the achievement of a significant reduction in the alarm rate, they deem that the changing of default settings and better education regarding cardiac monitors are insufficient to improve alarm system safety.

Scientific studies show that the quality of medical device alarms is unsatisfactory, and it affects quality of care and patient safety. One root cause is the poor quality of alarm-generating algorithms. Therefore, from a clinical perspective, major improvements in alarm algorithms are urgently needed [8].

To pursue this goal, different methods have been proposed and investigated for use in the alarm systems of medical devices, mostly from the fields of statistics and AI. Imhoff et al gave a brief overview of different methods, including statistical approaches (eg, improved data preprocessing, robust signal extraction, segmentation, median filter, statistical process control, and time series analysis for pattern detection, among others) and AI methods, such as knowledge-based approaches, knowledge discovery based on machine learning, neural networks, random forests, fuzzy logic, and Bayesian networks [8].

Regarding the methodological approaches to alarm management, Imhoff et al present the 4 areas in which alarms can be improved: (1) signal acquisition, that is, the interface between patient and medical devices; (2) alarm generation, that is, the algorithms that determine an alarm situation; (3) alarm validation, that is, determining whether the alarm is actually valid; (4) integration of multiple alarms, for example, from different devices, into 1 or few alarms [8].

Successful quality improvement approaches included alteration in default monitor presets, daily electrode change, alarm customization, alarm management education, change in policy, histogram-based pulse oximetry (SpO_2_), alarm tailoring, improved displays to aid in nurse-patient assignment, and the use of notification delays [10]. Notification delays are performed with a middleware situated between the alarming medical device and the clinicians’ receiver equipment such as a mobile phone. Several studies found that introducing alarm delays prior to the notification process could drop “false alarms” 25–67% [10]. Regarding the reduction of the total alarms, considering the effects of these interventions, alarm quantities decreased between 18.5% and as much as 89%, according to Winters et al [9].

The major contribution of our work described in this paper is mainly related to the integration/grouping of multiple alarms, where we present the application of a new alarm algorithm to reduce alarm fatigue. We evaluated our algorithm to reduce the total number of alarms through the use of real patient data. Our approach uses a notification delay approach to decide whether to deliver a unique notification to caregivers instead of several alarms for the same alarm situation. By using our system, we reduced the notifications received by the caregivers by up to 99.3% (582/586) of the total alarms generated.

#### A Step Before Reasoning: The Anomaly Detection and Alarm-Triggering Processes

Before presenting our reasoning mechanism, we outline important concepts of the monitoring process developed in our previous work related to coping with remote patient monitoring [11,12]. In this section, we illustrate a more formal model for the anomaly detection and the alarm-triggering processes that are used in our system.

The default functioning of our notification system is to notify a group of caregivers about anomalies detected in a patient’s vital signs. The anomaly detection process works through continuous monitoring of each patient’s vital signs. To verify if an anomaly occurs, the readings are evaluated against anomaly thresholds configured for each patient. If a reading for a patient is more than a maximum or less than a minimum threshold value, then the reading is considered to be anomalous and the system triggers an alarm that is sent to the health care team. The anomaly detection process and its related concepts such as anomalies, alarms, and notifications are defined in the next subsections.

#### Defining Thresholds and Anomalous Values

Anomaly thresholds for the sensors must be configured before starting to monitor a patient. A threshold is a minimum and maximum limit for a reading of a sensor S for a patient P, and an anomaly is a value either below or above those limits. An anomaly or anomalous value (AV) v∈*AV*(S,P) triggers an alarm that is sent to the health care team. The threshold value for sensor S connected to a patient P is designated threshold (S, P) and the minimum and maximum values are *v*_min(S, P) and *v*_max(S, P), respectively. We formally defined anomalies using set theory as shown later.

Let *AV*(S,P) be the set of values that represent patient P’s AV for the sensor S. Let us also consider that these values from S belong to the set of real numbers. The *AV*(S,P) set is formally defined as shown in Equation (1):

*AV*_S,P_={*v*| *v*∈R, *v*_min _S,P_>*v*>*v*_max _S,P_} (1)

Where:

1. The inequalities v<v_minS,P and v>v_maxS,P comprise the thresholds for sensor S and patient P.
2. v_minS,P∈R, which represents the minimum limit, that is, the value below which a sensor reading v is considered an AV.
3. v_maxS,P∈R, representing the maximum limit, that is, the value above which v is considered an anomaly.

We can define an anomaly detected by sensor as the function *An*(*v*)=*b* that maps real numbers into Booleans (f: R→ Boolean) where v∈R and is the value that represents a sensor reading, and *b*={true, false} as shown below.

An(v)=true, if v∈AVS,P; false, otherwise. (2)

#### Defining Alarm, Anomaly Detection, and Notification Events

In our system, we define the concepts of anomaly detection, alarm triggering, and notification in terms of events, which are represented as α, β, and μ, respectively.

The occurrence of an event α=“anomaly detected” means that the function *An*(v) assumes the value “true” at a given time defined as ANOMALY_DETECTION_TIME (*T*_α_). The event β=“alarm triggering,” in its turn, is defined as the action of triggering an alarm to indicate that an anomaly has been detected. The time when an event β occurs is referred as ALARM_TRIGGERING_TIME (*T*_β_). The third event we define in this section is μ=“notification.” μ is the action of sending a notification to a set of caregivers to inform them that an alarm has been triggered. The time when an event μ occurs is referred to as NOTIFICATION_TIME (*T*_μ_).

Associated with the occurrence of these events, we have the delays ALARM_TRIGGERING_DELAY (*D*_β_) and NOTIFICATION_DELAY (*D*_μ_), where *D*_β_ represents the delay between anomaly detection and its indication through an alarm triggering and *D*_μ_ is the delay between an alarm triggering and its notification to the caregivers. We show in Equations (3) and (4) how the delays *D*_β_ and *D*_μ_ are calculated according to the time at which the events α, β, and μ occur.

Dβ=Tβ−Tα (3)

Dμ=Tμ−Tβ (4)

We can summarize the abovementioned explanation in a more formal way through the event-trigger rules presented in Equations (5) and (6):

φ1: α→β (5)

φ2: β→μ (6)

where α, β, and μ are the events; the symbol “→” represents the action triggers; φ1 indicates that, when the event α occurs, the event β is automatically triggered after the delay *D*_β_; and φ2 indicates that event is automatically triggered *D*_μ_ time after β occurs.

The parameterization of the events α, β, and μ is defined as follows.

Α=<type, Tα > (7)

Β=<type, α, Tβ > (8)

Μ=<type, β, Tμ > (9)

where the parameter α for β event represents the event α; and the parameter β for μ event represents the event “alarm triggering” β.

#### Modeling Anomaly Detection, Alarm-Triggering, and Notification

**Figure 1:**
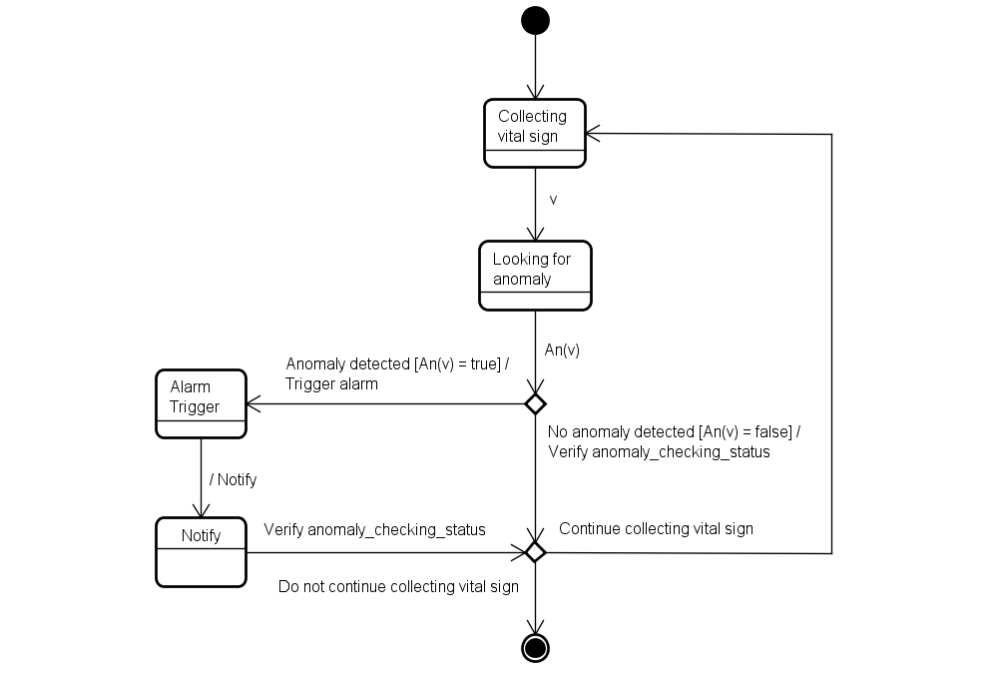
The state-transition machine showing the states involved in the anomaly detection, alarm-triggering, and notification processes.

To illustrate the anomaly detection, alarm-triggering, and notification processes, we present a state-transition diagram in Figure 1. This figure presents a visual representation of the following: (1) the possible states of the anomaly detection, alarm-triggering, and notification processes; (2) the events such as inputs that may result in transitions between states; and (3) the transitions between states. We also show the conditions an event requires to trigger a transition.

**Figure 2:**
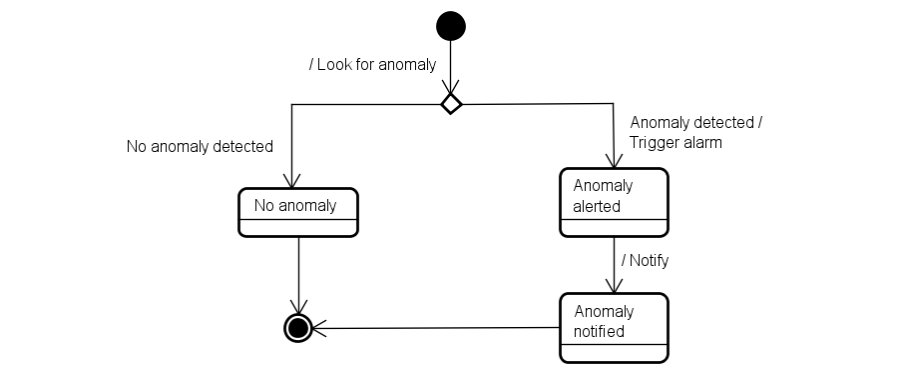
The state-transition machine showing the possible states for an anomaly.

To formalize the concept of an anomaly, we present, through the state-transition machine in Figure 2, the possible states for an anomaly. Figure 2 presents the current anomaly detection process, showing the 3 possible states of an anomaly: *no anomaly*, *anomaly alerted*, and *anomaly notified*. The interconnecting arrows represent the transitions between states, and the labels on the arrows represent the events that make the transitions occur.

Now that the basic concepts anomaly, alarm, anomaly detection, and notification needed for the reasoning process have been defined, in the next sections we present our reasoning model, system architecture, and algorithms.

#### Adding Reasoning to the System

In this section, we provide a brief description of how we apply a reasoning engine to the alarms generated by the monitoring devices being used to track a patient’s health status to minimize alarm fatigue. The software system contains a component that reads the vital signs (the reader) accompanied by a reasoning engine that decides how to notify the health care team. The reader can be set to ignore all the nonanomalous vital signs to focus only on the AV that can require attention from the caregivers’ team. An anomalous reading is then passed to the reasoning engine that decides how to handle the reading. For example, the reading could be used to cause an alarm to be triggered immediately because the patient’s situation is deemed critical; or readings could be accumulated as the situation is not critical but can be attended to within a certain time period.

#### The Alarm Fatigue-Aware Notification Model

**Figure 3:**
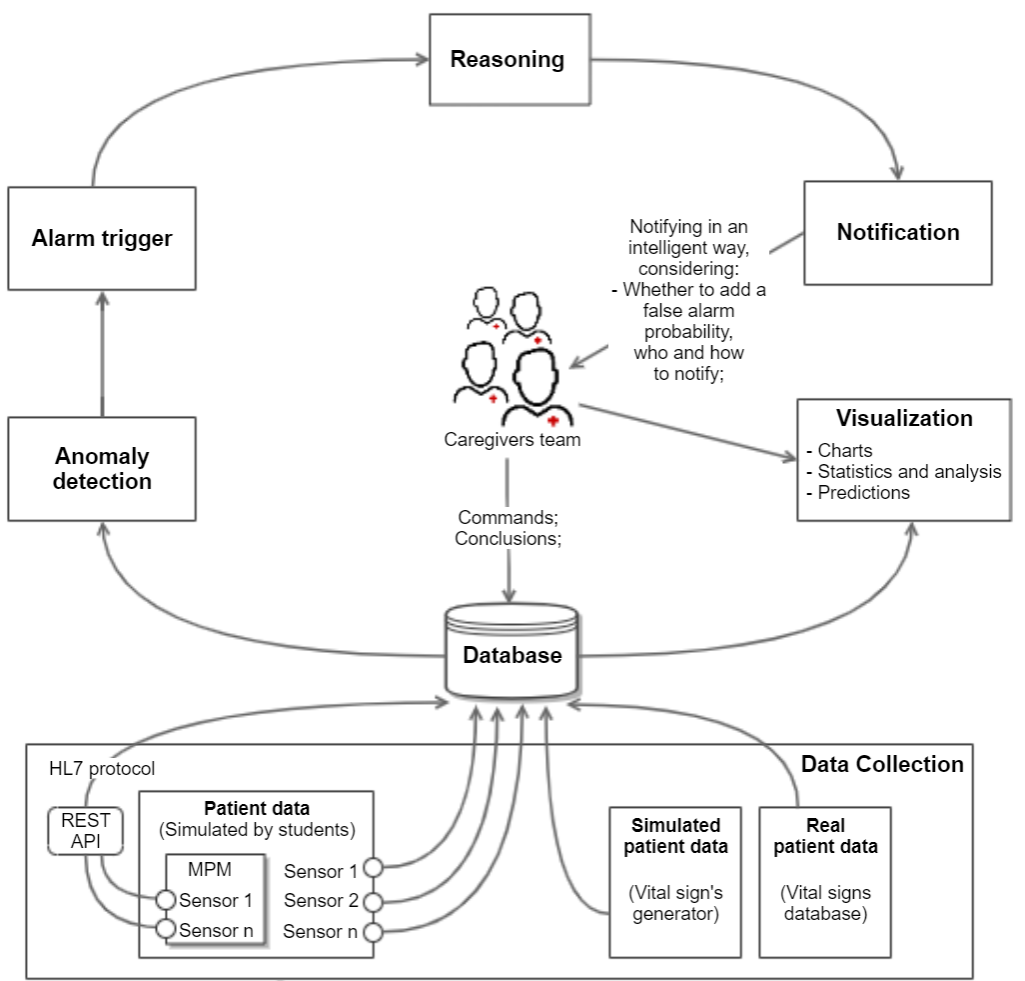
An architecture designed for health care systems that support patient monitoring and notification capabilities. MPM: Multi-parametric Monitor; API: Application Programming Interface.

Figure 3 presents our model designed to support reasoning algorithms that decide on the best approach to notify caregivers to avoid alarm fatigue. The reasoning algorithms, which are the focus of this research, decide the following: (1) whether to aggregate alarms, (2) whether to add a false alarm probability (FAP) label to the notification, and (3) who to notify within the group of caregivers.

### System Architecture

#### Updating the Anomaly Detection, Alarm-Triggering, and Notification Process Through the Addition of Reasoning

**Figure 4:**
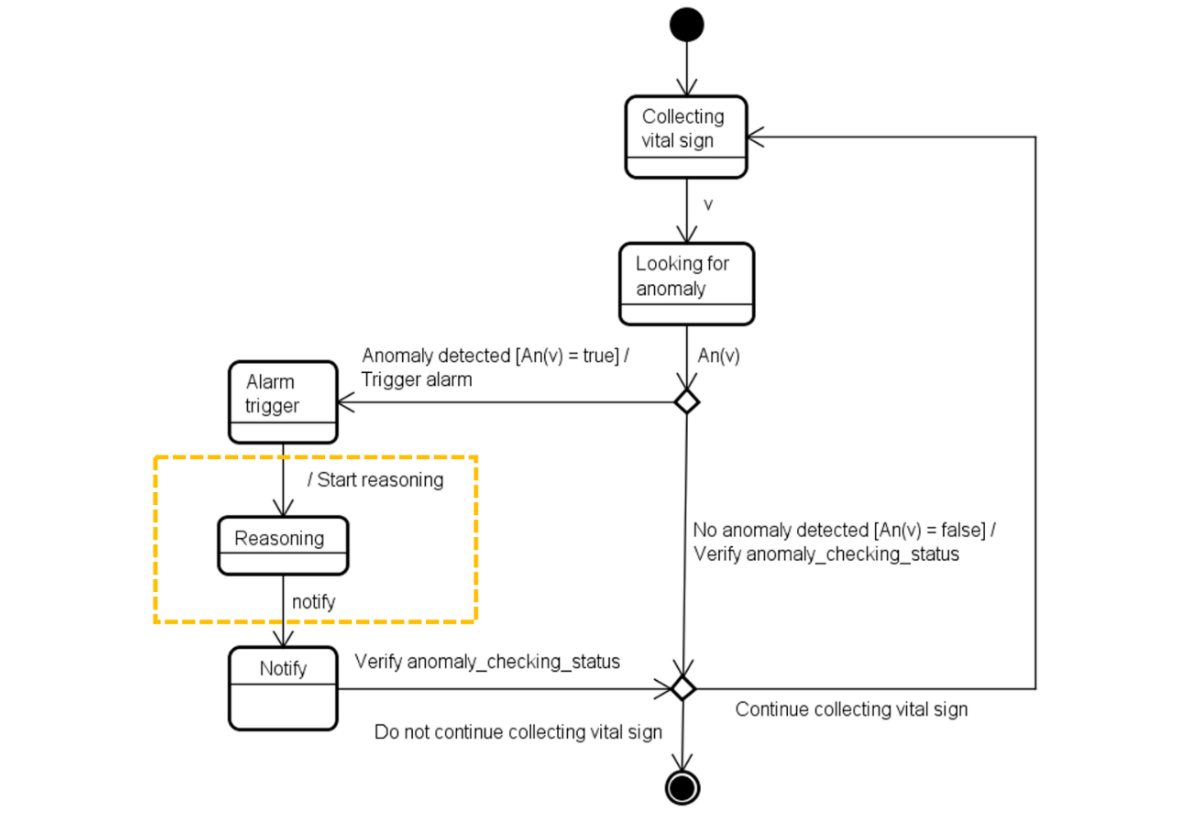
Illustration of the inclusion of the state “Reasoning” (inside the hatched rectangle) that determines when an alarm trigger(s) causes a notification.

Before presenting the reasoning algorithms, we show, in Figure 4, how the reasoning process interacts with the anomaly detection, alarm-triggering, and notification processes.

**Figure 5:**
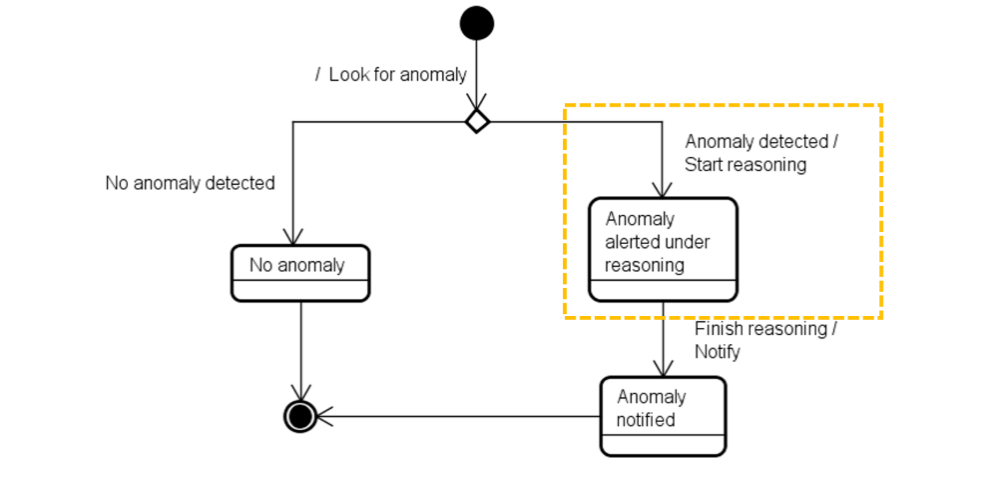
Illustration of the inclusion of the new state “Anomaly alerted under reasoning” (inside the hatched rectangle) as another possible state for an anomaly.

Figure 5 is an update of Figure 2 including information related to the reasoning activity.

**Figure 6:**
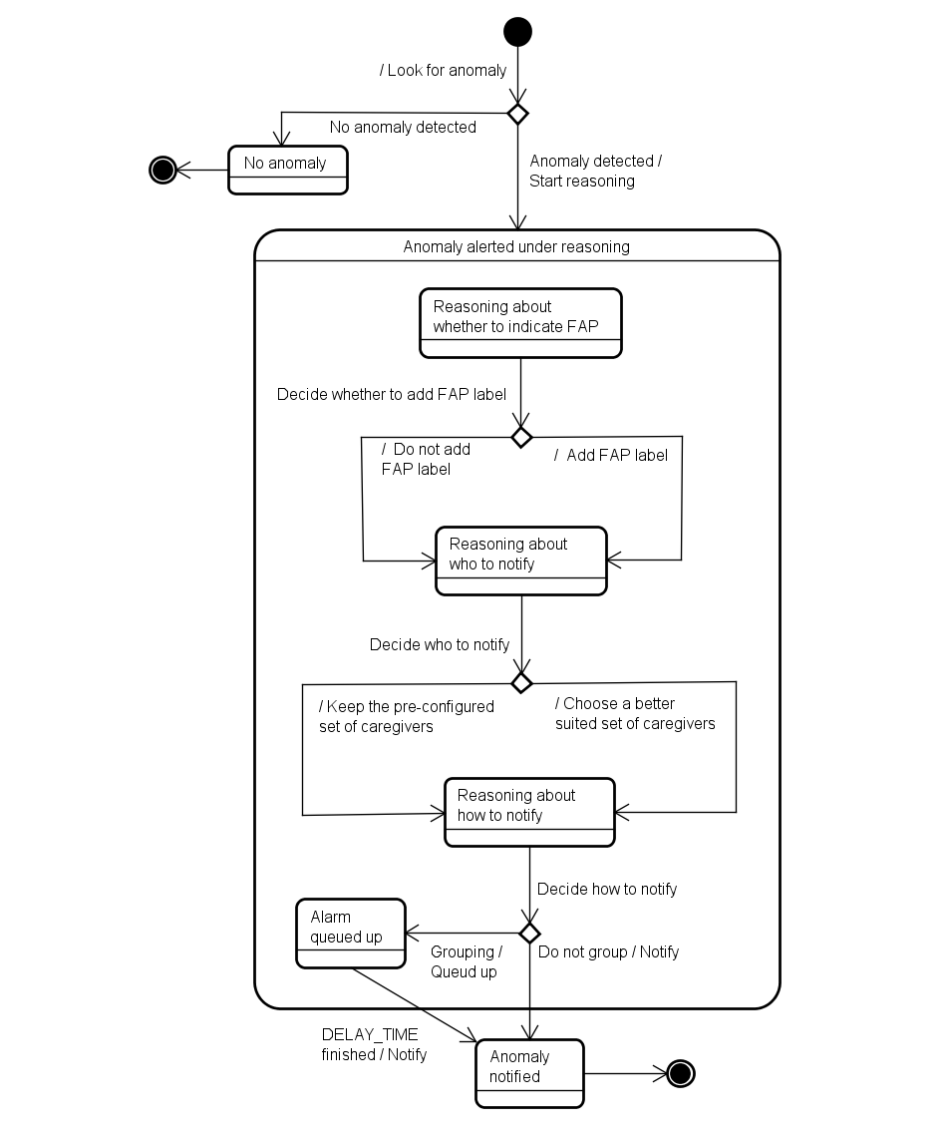
A high-level representation of the decision-making processes used during reasoning. FAP: false alarm probability.

To deal with the decision-making processes occurring during reasoning, we developed the Reasoner entity that is an instance of our reasoning algorithm. The Reasoner is responsible for managing the entire notification process. A high-level representation of the decision-making processes is shown in Figure 6.

#### Reasoning About How to Notify to Avoid Alarm Fatigue

In this section, the reasoning algorithm that is used to mitigate alarm fatigue is discussed. As has been mentioned, the default behavior of our anomaly detection process is to trigger an alarm every time an anomaly occurs, independent of circumstances. For example, a notification would occur even though the alarm was false, or a number of other alarms are happening. However, even though an alarm has been triggered by our patient monitoring system, the decision of how to notify the caregivers is decided by the Reasoner, using the following rule R1, which states:

- R1. Our system must limit to one of the number of notifications (of the same type for the same patient) that caregivers can receive within a defined period of time.

#### MINIMUM_NOTIFICATION_INTERVAL

We define Minimum_Notification_Interval (MNI) as the minimum interval of time between receiving 2 notifications by the caregivers. The R1 rule is only applied when we are considering notifications of the same type (TYPE_β) for the same patient P.

Let *μ_j_* and *μ_j_*_-1_ be 2 notifications of the same type for a given patient P. As shown in Equation (9), a notification can be formally defined as *μ*=<TYPE_*μ*, *T*_μ_, *P*>, and in this case we can assume that TYPE_*μ_j_* is equal to TYPE_*μ_j_*_-1_ and also that *P_j_* is equal to *P_j_*_-1_. The time *T_μ_*, at which the notification occurs, allows 2 notifications to be distinguished from each other. The MNI can be formally defined in terms of the notifications *μ_j_* and *μ_j_*_-1_ as shown below:

*T_μj_*−*T_μj_*_−1_>=MNI iff (TYPE_*μ_j_*=TYPE_*μ_j_*_−1_)∧(*P_j_*=*P_j_*_−1_) (10)

The MNI value must be configured for each patient individually based on patient’s context (both of the alarm sources, and patient’s criticality).

#### The Inputs for Our Reasoning Algorithm Related to a Notification

After explaining rule R1, we define the inputs (*I*) for our algorithm as follows:

- I1—CURRENT_ALARM_TRIGGERING_TIME (Tβr). Let βr be the current alarm that has been triggered and is involved in the reasoning process, so the algorithm can decide whether to add a delay to its delivery. The first input for our algorithm is Tβr, that is, the time when the alarm βr was triggered.
- I2—LAST_NOTIFICATION_TIME (Tμk). Let μk be the last notification (of the same type as βr) received by the caregivers. The second input for our reasoning algorithm is the time when caregivers received μk, which we represent as Tμk.

As we only consider here current alarms under reasoning and last notifications of the same type and from the same patient, we assume that the alarm types and patients are identical, that is, TYPE_β*_r_*=TYPE_*μ_k_* and *P_βr_*=*P_μk_*.

#### LAST_NOTIFICATION_PERIOD

Another definition is the Last_Notification_Period (LNP), which is the period of time between the 2 inputs for our reasoning as shown in Equation (11).

LNP=*T_βr_*−*T_μk_* (11)

#### The Outputs of Our Reasoning Algorithm Related to a Notification

We next define the outputs (O) for our reasoning algorithm as the 2 properties of notifications that can vary depending on the circumstances under which they occur:

O1—NOTIFICATION_DELAY (*D*_μ_). As discussed previously, in Equation (4), *D*_μ_ is the period of time between the *alarm triggering* event and the delivery of that notification to the caregivers.

O2—NOTIFICATION_DATA (DATAμ). DATAμ refers to the type of data a notification might contain, which depends on the context of the alarm-triggering process, and it might range from a single alarm β*_j_* to a set of alarms SET.

As much as possible, we try to keep the NOTIFICATION_DELAY at a minimum so as not to prejudice patient safety. However, to avoid alarm fatigue, the value for this property can range over an acceptable range of time defined as the BUFFERING_PERIOD, indicating that a DELAY_PERIOD (ε) might be added to the delivery time of the notification under specific conditions (defined in the next section). The BUFFERING_PERIOD is the period of time one or more alarms can be delayed (ie, be held in a buffer) before being delivered to caregivers. See Equation (12).

0<BUFFERING_PERIOD<MNI (12)

From Equation (12), we show that an alarm might need to be delayed up to a period equal to MNI. However, the BUFFERING_PERIOD specified for an alarm or a set of alarms should not surpass the value of MNI.

#### Defining the Grouping Criteria for Notification Delivery—When We Shall Put an Alarm Into Our Buffer

As we said previously, the Reasoner decides the way of delivering the alarm under reasoning (β*_r_*) by making choices about whether to add a delay ε to its delivery and whether to group β*_r_* with other alarms. To make these choices, the Reasoner must take into consideration our defined inputs (*T_βr_* and *T_μk_*). By analyzing these inputs, the Reasoner decides whether to queue the current alarm *β_r_*, based on the following grouping criteria:

- Criteria 1. A same-type alarm was already notified within the MNI.

If caregivers were already notified in the LNP, then the current alarm β*_r_* must be queued up into a buffer for the period BUFFERING_PERIOD. After BUFFERING_PERIOD has passed, β*_r_* is delivered along with other possible alarms in the buffer as a unique notification.

Just to clarify, when the circumstances for the alarms do not meet the abovementioned grouping criteria, a notification containing an individual alarm is sent to the caregivers as soon as an alarm has been triggered, that is, immediately after *T_βr_*.

As important as it is to avoid alarm fatigue, the Reasoner must handle the notification delivery process without compromising patient safety. In this case, the delay added to the notification delivery must not prejudice the requirements established regarding patient safety.

#### The Pseudocode for Our Reasoning Algorithm About How to Notify

The pseudocode for our reasoning algorithm about how to notify is shown in Textbox 1.

**The pseudocode for the reasoning about how to notify.**

DEFINE LNP, Tβ_r_, Tμ_k_, MNI;

// Receive Input CURRENT_ALARM_TRIGGERING_TIME Tβ_r_;

INPUT Tβ_r_;

// Receive Input LAST_NOTIFICATION_TIME Tμ_k_;

INPUT Tμ_k_;

// Calculate LNP

LNP=Tβ_r_−Tμ_k_;

// If LNP is equals to Tβ_r_ (meaning that no notification μ_k_ occurred to the patient in the last MNI-period) or LNP is higher than or equal to MNI (which means that a notification μ_k_ occurred more than MNI-period ago) then notify β_r_ immediately. Otherwise, put β_r_ into the buffer

If (LNP==Tβ_r_ ||LNP≥MNI) then

//There is no need for putting βr into the buffer. Notify it immediately

Notify(β_r_);

Else {

// We need to put βr into the buffer and deliver it after some delay

QueuedUp(β_r_)

// If βr is the first alarm been put into the buffer then {

Ìf (isAlarmTheFirstOneQueuedUp(β_r_)) then {

//Define buffer’s property STARTING_TIME as the time the alarm was triggered;

STARTING_TIME=Tβ_r;_

//Create a new thread for handling the buffer in parallel. This thread needs to

//control the BUFFERING_PERIOD (BP) for notifying caregivers after BP has passed

Create a new thread;

Start BUFFERING_TIME;

If BUFFERING_PERIOD has passed then

//Release the content of buffer to caregivers by wrapping the set of alarms

//(alarmsSet) into a single notification and sending it

Notify(alarmsSet);

}

}

## Methods

In this work, we present a new approach to cope with the alarm fatigue problem. Our proposed solution focuses on an automatic reasoner that is used to decide how to notify caregivers about anomalies detected by a patient monitoring system through a notification delay strategy.

To confirm the fulfillment of the main research goal, the experiment described next was conducted and results are tabulated in the Discussion section.

### Hypotheses

We defined the following hypotheses for our case study:

1. The caregivers should not receive more than one notification about the same type of anomaly for the same patient within the MNI.
2. Patient safety will not be compromised by the use of the reasoning algorithm about how to notify.

### Methodology

To illustrate the operation of our reasoning algorithm, we conducted 5 experiments to evaluate how the algorithm works under different scenarios, considering mainly the number of alarms generated in each experiment.

### Applications Settings

As shown in Table 1, to run an experiment, we need to define the following settings for our application scenarios:

- The number of wards occupied by patients (NUMBER_OF_WARDS).
- The number of patients being monitored (NUMBER_OF_PATIENTS) by a caregiver team.
- The number of sensors used during monitoring (NUMBER_OF_SENSORS).
- The interval in which the sensor readings are being monitored (SENSORS_READING_INTERVAL).
- The number of sensor readings (NUMBER_OF_READINGS). This information, along with the SENSORS_READING_INTERVAL, tells us how long the patients in our experiment are being monitored.

We also need to define the thresholds for each sensor and the MNI, considering each patient individually (Table 2). As has been mentioned earlier, the MNI is defined by taking into account both of the alarm sources, and the patient’s criticality to respect patient safety constraints. In our simulated environment, we defined the MNI value as 5 min for every patient and we assume the delivery of the type of anomalies triggered in our context (which are related to heart rate values) can be delayed up to this period without representing any danger for the patients.

All the inputs for our reasoning were provided through a vital signs streaming app, we developed for streaming vital signs retrieved from a dataset comprising real patient data recorded from patients undergoing anesthesia at the Royal Adelaide Hospital. The dataset provides clinical anesthesia monitoring data from 32 entire surgical cases, including a wide range of vital signs variables, such as electrocardiograph, pulse oximeter, capnograph, noninvasive arterial blood pressure monitor, airway flow, and pressure monitor, and in a few cases, a Y-piece spirometer, an electroencephalogram monitor, and an arterial blood pressure monitor [10]. The monitoring data were collected using Philips IntelliVue MP70 and MP30 patient monitors and Datex-Ohmeda Aestiva/5 anesthesia machines. In this dataset, a single stream of raw monitoring data was recorded in a comma-separated values (CSV) text file format at a sampling resolution of 10 milliseconds [10].

We evaluated our algorithm by using data that we selected from 3 out of the 32 surgical cases in the dataset (cases 04, 07, and 14). Experiment 1 was conducted using data from case 4, while, in experiment 2, we utilized data from case 14, and, finally, experiments 3-5 were executed using data from case 7. In all the experiments, we utilized the version of processed data available in the CSV format for monitoring patients based on their heart rate parameter at 1-second intervals (our algorithm uses this frequency instead of the 10-millisecond sampling resolution available at the dataset). However, the number and type of vital signs used in every experiment could vary to simulate other configurations for sensors and monitoring devices in an ICU.

To define when a given heart rate reading represented an anomalous value that should trigger an alarm, we defined the thresholds in Table 2 for each patient.

**Table 1:** Defining the configuration for our 5 experiments.

Number of wards	Number of patients	Number of sensors	Sensors reading interval (ms)	Number of readings
1	1	1	1000	60,000

**Table 2:** Defining the anomaly thresholds of heart rate sensor for each patient.

Experiment	Patient_ID	Min_heart rate	Max_ heart rate
1	1	60	100
2	2	55	100
3	3	50	105
4	4	50	100
5	5	50	102

## Results

### Application Details—Technologies Utilized

**Figure 7:**
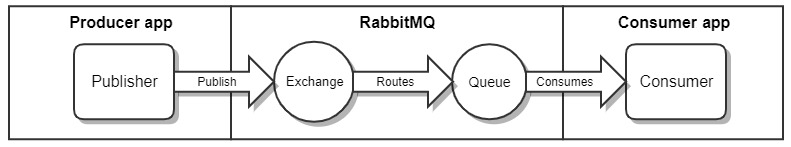
Basic concepts and information flow in RabbitMQ.

The application was developed in the Java language along with the use of the RabbitMQ [13] message broker. RabbitMQ is an open-source message broker that accepts, stores, and forwards messages. The basic concepts behind this technology are Queue, Producer, and Consumer (Figure 7). A Queue is essentially a large message buffer that stores the messages, while a Producer and a Consumer are both user applications. The former is a program in charge of sending messages to the queue through the exchanges, and the latter consists of a program that receives messages from the queue. A program can be both a Producer and a Consumer at the same time [13].

As can be seen from Figure 7, a broker receives messages from publishers (producers) and routes them to the consumers. The information flow involved in this process occurs in 2 steps, described as follows:

- Step 1. The producers send messages to exchanges that act by distributing messages to queues using rules called bindings.
- Step 2. The broker either delivers messages to consumers subscribed to queues or consumes pull messages from queues on demand.

In this application, we used the Advanced Message Queuing Protocol 0-9-1 Java client provided by RabbitMQ, which is an open and general-purpose protocol for messaging.

Owing to the high volume of notifications we are dealing with in our application, we decided to utilize a solution that could take care of the nonfunctional requirements of our system. By using a solution to handle problems related to scalability and safety, we could focus on the functional requirements of our application. Therefore, we decided to use the RabbitMQ to meet the high availability, throughput, and scale requirements of our application domain. This message broker solution offers features related to data safety such as reliable delivery, which means it can ensure that messages are always delivered, even encountering failures such as network failures and consumer application failures [13].

### Explaining How Our Application Works

In a high abstraction level, the main idea of this app is to have an application that sends alarms to a broker that routes them to a consumer app that represents the receiving of these alarms by the health care team.

**Figure 8:**
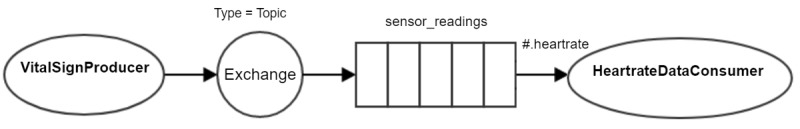
RabbitMQ scheme utilized in our application.

We chose the type of exchange called *topic* for routing the messages. The topic exchange routes messages to one or many queues based on matchings between a message routing key and the pattern that is used to bind a queue to an exchange. We declared one queue named *sensor_readings* to where the publisher sends the data and the consumer receives data. We also declared the binding key for our consumer (ie, the class that is consuming heart rate data) as *#.heartrate* (Figure 8).

The routing key is defined based on the pattern <patientID>.<heartrateValue>. For example, we could have a routing key as *16.88*, representing a patientID=16 and heartrateValue=88.

The notifications sent to health providers are created based on this message. In this case, the final notification received by nurses contains information related to the patient, such as identification, location, and vital signs.

### Application Modeling—Class Diagram

**Figure 9:**
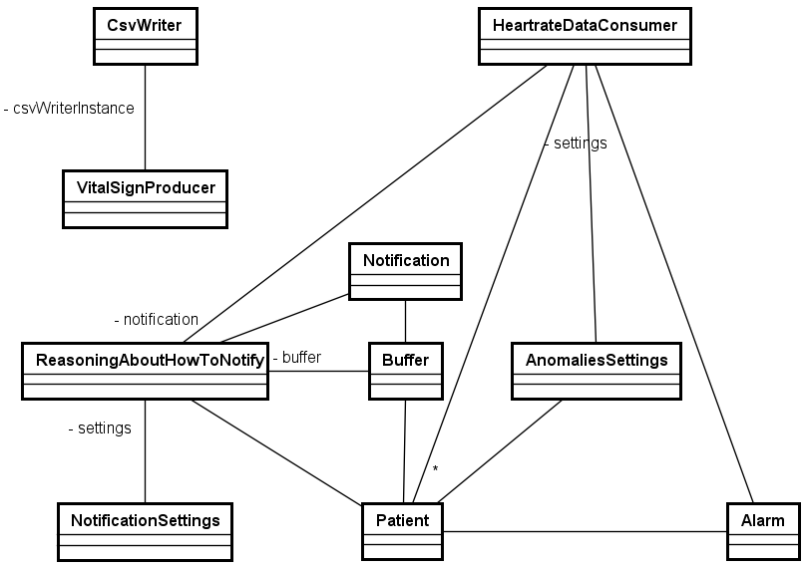
The class diagram for our application, where the consumer application monitors a specific vital sign based on the anomalies settings defined for each patient.

In Figure 9, as can be seen from the class diagram for our application, the consumer application monitors a specific vital sign based on the anomalies settings defined for each patient. The consumer app invokes the reasoning mechanism through the ReasoningAboutHowToNotify class, which knows how to notify based on the defined notifications settings (eg, the MNI value configured for each patient).

**Figure 10:**
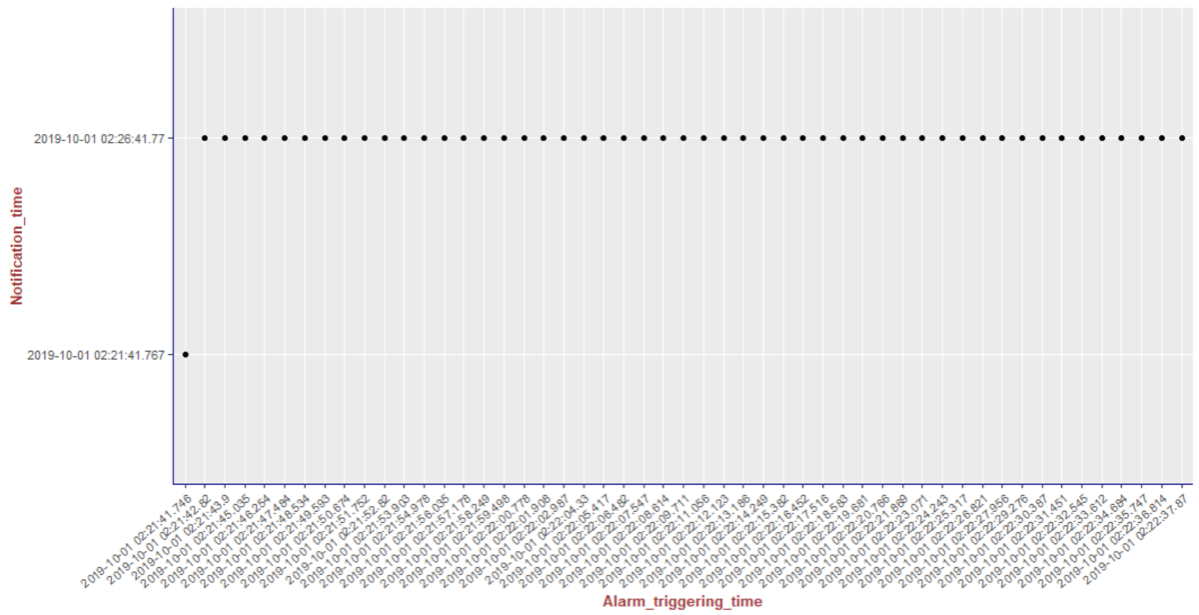
Illustration of the results of the alarm triggering and delivery processes related to the patient monitored in our experiment 5 (PatientID=5).

**Figure 11:**
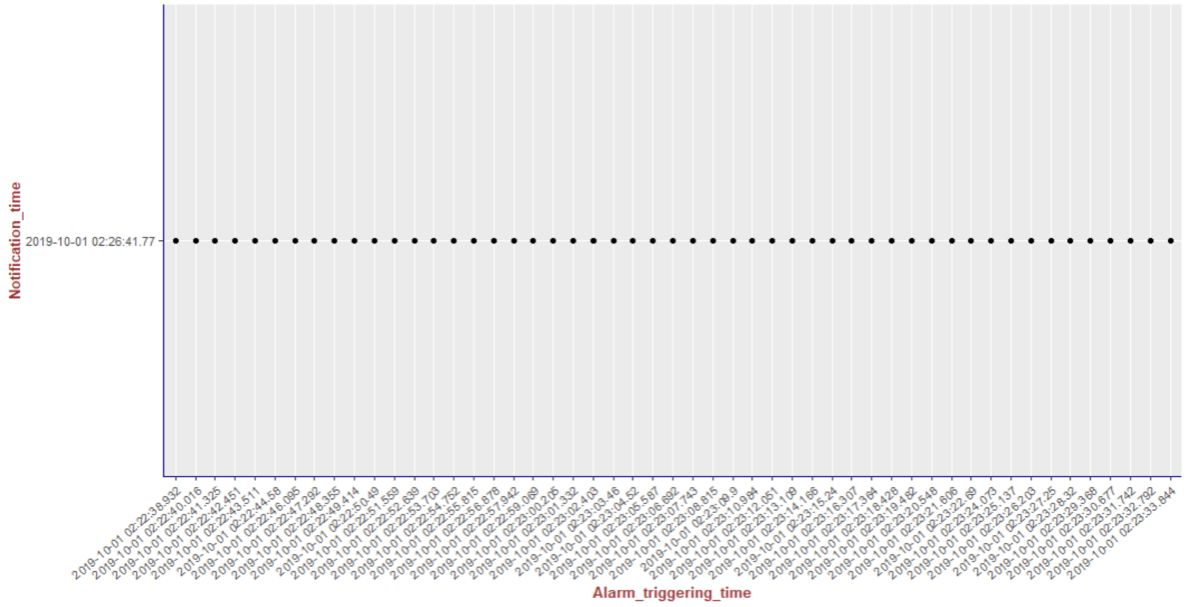
Illustration of the results of the alarm triggering and delivery processes related to the patient monitored in our experiment 5 (PatientID=5).

**Figure 12:**
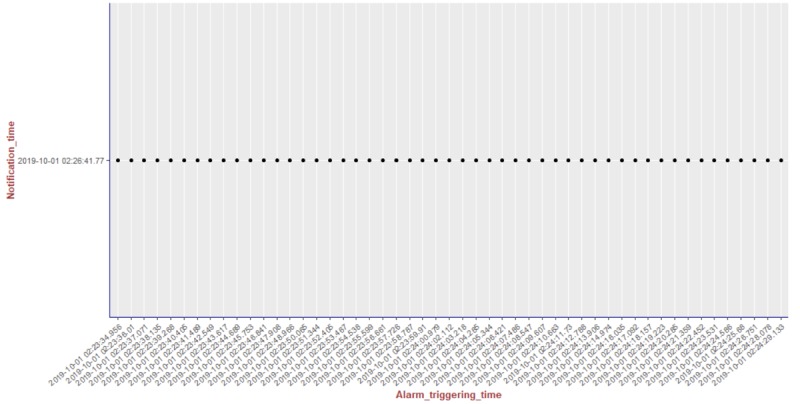
Illustration of the results of the alarm triggering and delivery processes related to the patient monitored in our experiment 5 (PatientID=5).

**Figure 13:**
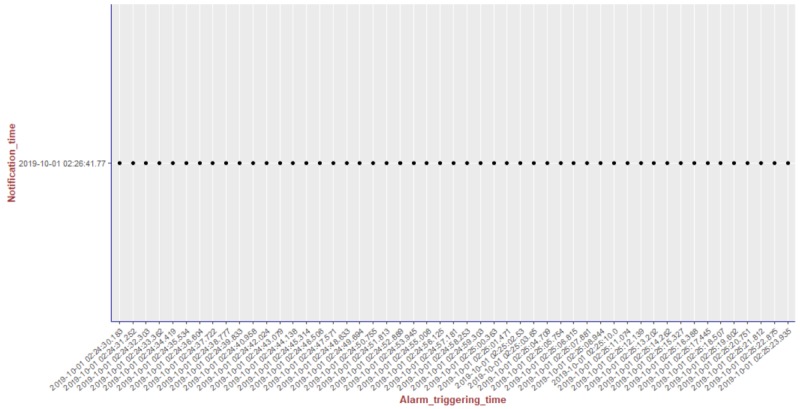
Illustration of the results of the alarm triggering and delivery processes related to the patient monitored in our experiment 5 (PatientID=5).

We present the results of our algorithm by using graphs we generated using the R language and the ggplot2 [14] library. The graphs shown in Figures 10-13 illustrate the delivery process of all notifications related to the patient monitored in experiment 5 (PatientID=5). We show whether the algorithm decided to deliver an alarm immediately or after a delay by grouping alarms to deliver them together.

To better visualize the results of experiment 5 through the graphs, we split the output data of our algorithm for this experiment (comprising a total of 204 alarms) into 4 pieces of data containing 51 alarms each. Thus, we plot each piece of data into a graph, showing the alarm triggering time through the *x*-axis and the notification time on the *y*-axis. As can be seen from Figure 10, the occurrence of the first notification (NotificationID=1) of an alarm of heart rate for patient 5 happened at the notification time *2019-10-01 02:21:41.767*, that is, almost immediately after the occurrence of the first alarm (that happened at the alarm triggering time *2019-10-01 02:21:41.746*). Following the strategy of our reasoning algorithm, the next notification of an alarm of heart rate for this patient should not be received by the caregivers before MNI. As in this experiment MNI corresponds to 5 min, the timestamp for the next delivery of a heart rate alarm related to patient 5 should occur at least 5 min after *2019-10-01 02:21:41.767*. As can be seen in Figure 10, the next heart rate alarms for patient 5 were held in the alarms buffer and delivered together at the timestamp *2019-10-01 02:26:41.77* as a unique notification (NotificationID=2) with a delay of approximately 5 min.

**Figure 14:**
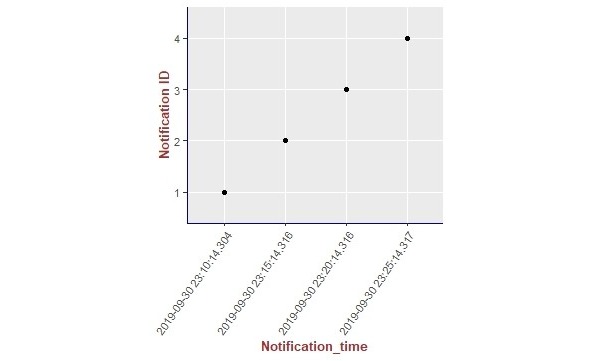
Illustration of the results of the delivery process related to all our experiments.

**Figure 15:**
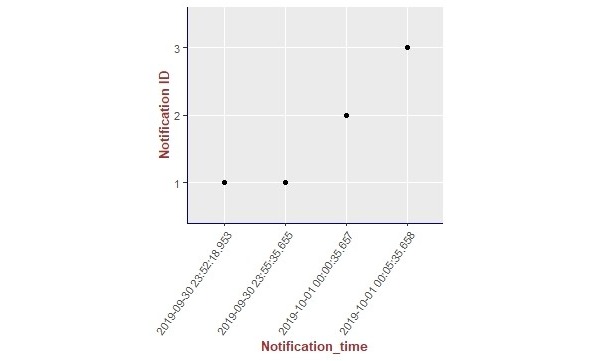
Illustration of the results of the delivery process related to all our experiments.

**Figure 16:**
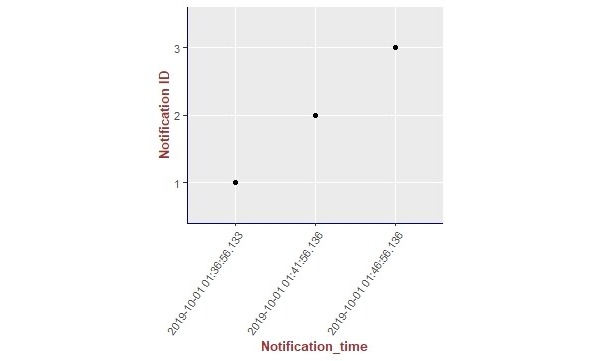
Illustration of the results of the delivery process related to all our experiments.

**Figure 17:**
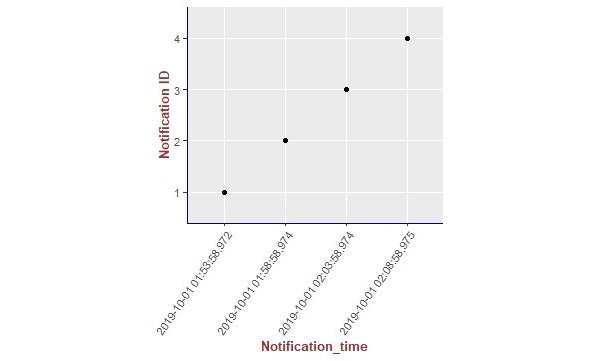
Illustration of the results of the delivery process related to all our experiments.

**Figure 18:**
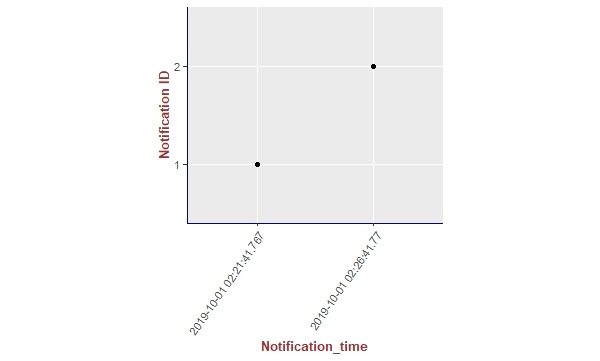
Illustration of the results of the delivery process related to all our experiments.

Figures 14-18 illustrates the results of the delivery processes related to all patients monitored in our experiments (PatientID=1,2,3,4, and 5, respectively).

We show the results for all of our experiments summarized in Table 3, where we can compare the number of alarms triggered by our system in each experiment with the number of notifications delivered to the caregivers.

**Table 3:** Results of our experiments to evaluate our reasoning algorithm about how to notify caregivers considering the reduction of the number of notifications received by them.

Experiment	Heart rate alarms, n	Heart rate notifications,	Notifications in relation to the total of alarms, %	Reduction in alarms received, %
1	407	4	0.9	99.0
2	423	3	0.7	99.2
3	308	3	0.9	99.0
4	586	4	0.6	99.3
5	204	2	0.9	99.0

## Discussion

The first hypothesis we want to evaluate with this case study says that the caregivers should not receive more than one notification about the same type of anomaly for the same patient within the defined MNI. By executing our reasoning algorithm throughout the experiments, we saw that hypothesis 1 holds for all of them, as within all the occurrences of notifications for each patient, there is no occurrence of a notification of the same type within the defined MNI. We support this affirmation by presenting, in Figures 14-18, a summary of the results from our experiments using graphs containing all notifications that occurred in each experiment. As can be seen, considering all experiments, there was no occurrence of delivery of notifications of the same type for the same patient that happened before the specified delay, that is, the MNI value of 5 min.

The second hypothesis that says that patient safety will not be compromised by the use of the reasoning algorithm about how to notify also holds, as the notification interval (MNI) we defined is no longer than 5 min. This means that a group of alarms that are happening to a given patient can be held in a buffer for, at most, 5 min before the buffer is fully released to the caregivers as a unique notification. However, in order not to prejudice patient safety, the first occurrence of an alarm is always delivered to the caregivers immediately after its occurrence. In this case, only the next occurrences of the alarms are delivered to caregivers with the addition of a given delay.

In Table 3, we made a comparison between the number of alarms triggered by our system and the number of notifications delivered to the caregivers, in each experiment. These results show that the reduction of the notifications received by the caregivers can be up to 99.3% (582/586) of the total of alarms, with a mean of 99.17% (1912/1928) of reduction in the number of total alarms, considering all the experiments.

According to Winters et al, nearly all studies assume that a reduction in the number of total alarms and/or false alarms will reduce alarm fatigue [9]. Thus, by presenting these results, we expect that our algorithm can be used as a useful strategy for avoiding alert fatigue. We also expect our approach can be useful for helping to prevent its negative consequences, such as disruption of patient care, disabling of alarm systems by staff, reduction in responding, lack of caregiver response, and real events being less likely to be acted on, among others.

In future work, we are planning to extend our approach to reason about whether to notify the caregivers’ team with an indication of a FAP. The idea is to provide a reliable classification system in which caregivers may trust so the FAP label added to the notification can help them prioritize their work, especially when they are under alarm fatigue conditions.

Other important future work focuses on how to use reasoning to decide whom to notify within the group of caregivers, considering their specialization level, degree of experience, availability, geolocation, and current workload conditions.

Note that our system is experimental and does not consider security, something that needs to be taken very seriously in an operational health care alarm system.

## Abbreviations

AI: artificial intelligence
AV: anomalous values
FAP: false alarm probability
ICU: intensive care unit
IT: information technology
LNP: last notification period
MNI: minimum notification interval

## References

[1] Cvach M (2012). Monitor alarm fatigue: an integrative review. Biomed Instrum Technol.

[2] Harris P, Zègre-Hemsey JK, Mammone T, Schindler D, Salas-Boni R, Bai Y, Tinoco A, Ding Q, Hu X, Drew (2014). Insights into the problem of alarm fatigue with physiologic monitor devices: a comprehensive observational study of consecutive intensive care unit patients. PLoS One.

[3] Tanner T (2013). The problem of alarm fatigue. Nurs Womens Health.

[4] Jones K (2014). Alarm fatigue a top patient safety hazard. Can Med Assoc J.

[5] Shanmugham M, Strawderman L, Babski-Reeves K, Bian L (2018). Alarm-related workload in default and modified alarm settings and the relationship between alarm workload, alarm response rate, and care provider experience: quantification and comparison study. JMIR Hum Factors.

[6] Sowan AK, Gomez TM, Tarriela AF, Reed CC, Paper BM (2016). Changes in default alarm settings and standard in-service are insufficient to improve alarm fatigue in an intensive care unit: a pilot project. JMIR Hum Factors.

[7] Karnik A, Bonafide CP (2015). A framework for reducing alarm fatigue on pediatric inpatient units. Hosp Pediatr.

[8] Imhoff M, Kuhls S, Gather U, Fried R (2009). Smart alarms from medical devices in the OR and ICU. Best Pract Res Clin Anaesthesiol.

[9] Winters BD, Cvach MM, Bonafide CP, Hu X, Konkani A, O'Connor MF, Rothschild JM, Selby NM, Pelter MM, McLean B, Kane-Gill SL, Society for Critical Care Medicine Alarm and Alert Fatigue Task Force (2018). Technological distractions (part 2): a summary of approaches to manage clinical alarms with intent to reduce alarm fatigue. Crit Care Med.

[10] Liu D, Görges M, Jenkins SA (2012). University of Queensland vital signs dataset: development of an accessible repository of anesthesia patient monitoring data for research. Anesth Analg.

[11] Fernandes CO, Lucena CJ (2017). A software framework for remote patient monitoring by using multi-agent systems support. JMIR Med Inform.

[12] Fernandes CO, de Lucena CJ, de Souza e Silva D (2017). Smart Depth of Anesthesia Monitoring With EEG Sensors and Agent-based Technology. 2017 IEEE SmartWorld, Ubiquitous Intelligence & Computing, Advanced & Trusted Computed, Scalable Computing & Communications, Cloud & Big Data Computing, Internet of People and Smart City Innovation.

[13] RabbitMQ.

[14] Hadley W (2009). ggplot2 - Elegant Graphics for Data Analysis.

